# *Artemisia absinthium* L. Aqueous and Ethyl Acetate Extracts: Antioxidant Effect and Potential Activity In Vitro and In Vivo against Pancreatic α-Amylase and Intestinal α-Glucosidase

**DOI:** 10.3390/pharmaceutics14030481

**Published:** 2022-02-22

**Authors:** Asmae Hbika, Nour Elhouda Daoudi, Abdelhamid Bouyanzer, Mohamed Bouhrim, Hicham Mohti, El Hassania Loukili, Hamza Mechchate, Rashad Al-Salahi, Fahd A. Nasr, Mohamed Bnouham, Abdelhamid Zaid

**Affiliations:** 1 Laboratory of Applied Chemistry and Environment, Team Applied Analytical Chemistry of Materials and Environment Faculty of Sciences, Mohammed First University, Oujda 60000, Morocco; asmaae.hbika@gmail.com (A.H.); bouyanzer@yahoo.fr (A.B.); e.loukili@ump.ac.ma (E.H.L.); 2 Laboratory of Bioresources, Biotechnology, Ethnopharmacology and Health, Department of Biology, Faculty of Sciences, University Mohamed First, Boulevard Mohamed VI, Oujda 60000, Morocco; nourelhoudada95@gmail.com (N.E.D.); mohamed.bouhrim@gmail.com (M.B.); m.bnouham@yahoo.fr (M.B.); 3 Laboratory of Management and Valorization of Natural Resources, Department of Biology, Faculty of Sciences, Moulay Ismail University, BP 11201 Zitoune, Meknes 50070, Morocco; hicham.mohti@gmail.com (H.M.); a.zaid@umi.ac.ma (A.Z.); 4 Laboratory of Inorganic Chemistry, Department of Chemistry, University of Helsinki, FI-00014 Helsinki, Finland; 5 Department of Pharmaceutical Chemistry, College of Pharmacy, King Saud University, Riyadh 11451, Saudi Arabia; ralsalahi@ksu.edu.sa; 6 Department of Pharmacognosy, College of Pharmacy, King Saud University, Riyadh 11451, Saudi Arabia; fnasr@ksu.edu.sa

**Keywords:** *Artemisia absinthium*, antioxidant activity, phenolic compounds, hyperglycemia, pancreatic α-amylase, intestinal α-glucosidase

## Abstract

*Artemisia absinthium* L. is one of the plants which has been used in folk medicine for many diseases over many centuries. This study aims to analyze the chemical composition of the *Artemisia absinthium* ethyl acetate and its aqueous extracts and to evaluate their effect on the pancreatic α-amylase enzyme and the intestinal α-glucosidase enzyme. In this study, the total contents of phenolic compounds, flavonoids, and condensed tannins in ethyl acetate and the aqueous extracts of *Artemisia absinthium* leaves were determined by using spectrophotometric techniques, then the antioxidant capacity of these extracts was examined using three methods, namely, the DPPH (2, 2-diphenyl-1picrylhydrazyl) free radical scavenging method, the iron reduction method FRAP, and the β-carotene bleaching method. The determination of the chemical composition of the extracts was carried out using high-performance liquid chromatography—the photodiode array detector (HPLC-DAD). These extracts were also evaluated for their ability to inhibit the activity of the pancreatic α-amylase enzyme, as well as the intestinal α-glucosidase enzyme, in vitro and in vivo, thus causing the reduction of blood glucose. The results of this study showed that high polyphenol and flavonoid contents were obtained in ethyl acetate extract with values of 60.34 ± 0.43 mg GAE/g and 25.842 ± 0.241 mg QE/g, respectively, compared to the aqueous extract. The results indicated that the aqueous extract had a higher condensed tannin content (3.070 ± 0.022 mg EC/g) than the ethyl acetate extract (0.987 ± 0.078 mg EC/g). Ethyl acetate extract showed good DPPH radical scavenging and iron reduction FRAP activity, with an IC_50_ of 0.167 ± 0.004 mg/mL and 0.923 ± 0.0283 mg/mL, respectively. The β-carotene test indicated that the aqueous and ethyl acetate extracts were able to delay the decoloration of β-carotene with an inhibition of 48.7% and 48.3%, respectively, which may mean that the extracts have antioxidant activity. HPLC analysis revealed the presence of naringenin and caffeic acid as major products in AQE and EAE, respectively. Indeed, this study showed that the aqueous and ethyl acetate extracts significantly inhibited the pancreatic α-amylase and intestinal α-glucosidase, in vitro. To confirm this result, the inhibitory effect of these plant extracts on the enzymes has been evaluated in vivo. Oral intake of the aqueous extract significantly attenuated starch- and sucrose-induced hyperglycemia in normal rats, and evidently, in STZ-diabetic rats as well. The ethyl acetate extract had no inhibitory activity against the intestinal α-glucosidase enzyme in vivo. The antioxidant and the enzyme inhibitory effects may be related to the presence of naringenin and caffeic acid or their synergistic effect with the other compounds in the extracts.

## 1. Introduction

The variety of molecules constituting aromatic and medicinal plants raises interest in several different fields, especially in the pharmaceutical industry; in fact, numerous food supplements and drugs are manufactured from plants. For this reason, various studies have been oriented towards the characterization and identification of new bioactive substances that can improve pharmaceutical production. *Artemisia absinthium* L. (*A. absinthium*) is among the plants with several pharmaceutical properties. This plant belongs to the *Asteraceae* family, and it is popularly known as wormwood in the United Kingdom and absinthe in France; it has been utilized as a medicinal plant in Europe, Asia, the Middle East, and North of Africa [1]. Well known for its many biological properties, including as an antispasmodic [2], antimalarial [3] antipyretic [4], antitrypanosomal [5], acaricidal [6] antibacterial [7], anti-inflammatory [8], antioxidant [9], antidepressant [10], and also as a treatment for chronic fever [11]. *A. absinthium* is being used in face serums, essences, masks, shampoos, and other cosmetology products [12]. It is used in the food industry, and it is also widely used as a key aromatic ingredient [13] in the manufacture of some alcoholic drinks and also as an ingredient in absinthe [14].

The Egyptian Ebers Papyrus from about 1552 BC and the Old Testament of the Bible both contain references to *A. absinthium* [15]. Archaeologists have found tablets with cuneiform characters referring to *A. absinthium* that probably originated in the Babylonian civilizations [16]. The Greek mathematician and philosopher Pythagoras of Samos recommended the use of absinthe as a treatment for the pains of childbirth (569–475 BC). Hippocrates (460–377 BC) used *A. absinthium* extracts for the treatment of rheumatism and menstrual pain, and he also recommended it for jaundice [16]. In Ethiopia, *A. absinthium* is used in rituals called Atete; it was known as ariti, and was also used for the treatment of non-infectious and infectious diseases such as malaria, helminths, and animal injuries [10,17]. *A. absinthium* has been used as a treatment for gastric irritation since the Middle Ages, since it acts as an aromatic bitter and is believed to stimulate the acid secretion and bile production in small doses. In the Middle Ages, *A. absinthium* was employed as both a vermifuge and a purge, and it became known as a “general remedy for all diseases”. Pure essential oil is very toxic and can lead to the death of those who consume it. *A. absinthium* has been used in the treatment of swelling, chronic fever, inflammation of the liver, and also as a stimulant and tonic [18]. For centuries, it has been used in the treatment of intermittent and chronic fevers in indigenous medicine [19]. For centuries, this plant has been used infused with tea by the Moroccan people in winter.

Diabetes mellitus is among the most common diseases in the world. Indeed, in 2017, 325 million people suffered from type 2 diabetes, and the number of cases is progressively increasing [20]. In 2006, type 2 diabetes was responsible for about 5% of global deaths, according to World Health Organization. The incidence of diabetes is increasing daily, and it is proposed that the number of cases will around 552 million by the year 2030 [21]. Diabetes mellitus is known as a chronic metabolic disorder defined by hyperglycemia, with disturbances in the metabolism of carbohydrates, proteins, and lipids. This disturbance is either due to an extreme deficiency in insulin synthesis, which is called type 1 diabetes, or primarily by insulin resistance, which is called type 2 diabetes. [22]. To achieve normal blood glucose levels in people with type 2 diabetes mellitus, oral hypoglycemic agents or insulin are required. However, the use of these drugs has been shown to have limited efficacy and is combined with undesirable side effects, which has led to an increasing focus on the use of herbs to limit these effects [23,24]. Pancreatic α-amylase and intestinal α-glucosidase are enzymes that decompose the long-chain carbohydrates that catalyze the cleavage of the starch to the disaccharide and the disaccharide to the glucose, respectively, and are therefore effective in retarding the absorption of glucose into the bloodstream, resulting in a hypoglycemic effect [25]. Pancreatic α-amylase is an enzyme that comes from the salivary glands and the pancreas. It is a species that is responsible for the digestion of carbohydrates, thanks to its ability to catalyze the initial hydrolysis of starch by acting on the internal bonds of carbohydrates α-D1,4. Pancreatic α-amylase is an enzyme that can break down starch into dextrins, maltotriose, and maltose [26]. Intestinal α-glucosidase is an enzyme of the intestinal brush border. It catalyzes the release of absorbable monosaccharides, such as glucose, from the substrate, ultimately facilitating absorption by the small intestine [27]. Natural phenolics have been shown to reduce the activity of enzymes such as pancreatic α-amylase and intestinal α-glucosidase [28]. It is estimated that about 800 plants may have anti-diabetic properties [29]. In the traditional treatment of diabetes, the plants belonging to the *Asteraceae* family are the most widely documented [22]. Li et al. [30] showed that *A. absinthium* exhibits antidiabetic activity in diabetic humans, with no significant effect on lipid profiles. Additionally, Daradka et al. [31] showed that the ethanolic extract of *A. absinthium* has a hypoglycemic activity in rats with alloxan-induced diabetes, exhibiting biosafety, with the improvement of associated biochemical parameters and the prevention of serious reduction in body weight. These studies led us to the study of the antidiabetic effect of this plant, using other methods and other solvents of extraction, as well as other techniques, and also to elucidate some mechanisms of action that explain the antidiabetic effect of this plant, targeting the digestive enzymes related to the digestion of carbohydrates (α-amylase and α-glucosidase). The analysis of *A. Absinthium* showed the presence of chemical compounds such as polyphenolic compounds [32] and flavonoids [33]. In studies on the bioactive compounds of *A. Absinthium*, flavonoids, terpenoids, coumarins, sterols, tannins, carotenoid glycosides, and bitter principles have been cited. [10,34]. In this work, we determined the phenolic compounds, condensed tannins, and flavonoids, as well as the chemical compounds in the extracts by HPLC-DAD; we also evaluated the extract antioxidant activities using DPPH, FRAP, and β-carotene bleaching methods. In addition, we studied the inhibitory activity of the extract against pancreatic α-amylase and intestinal α-glucosidase by using various approaches in vivo and in vitro.

## 2. Materials and Methods

### 2.1. Chemicals and Reagents

Phlorizin dehydrates and the pancreatic α-amylase enzyme were obtained from Sigma-Aldrich, USA; intestinal α-glucosidase, acarbose, streptozotocin, and dinitrosalicylic acid were obtained from Sigma-Aldrich in China. Anhydrous D (+) glucose was sourced from Riedel-de Haen, Germany. The solvents used in this work are of analytical grade and have been provided from Honeywell Riedel-de Haen. All chemicals were high-quality analytical chemicals and were used exactly as they were obtained from Merck.

### 2.2. Plant Material and Extraction

**Figure 1:**
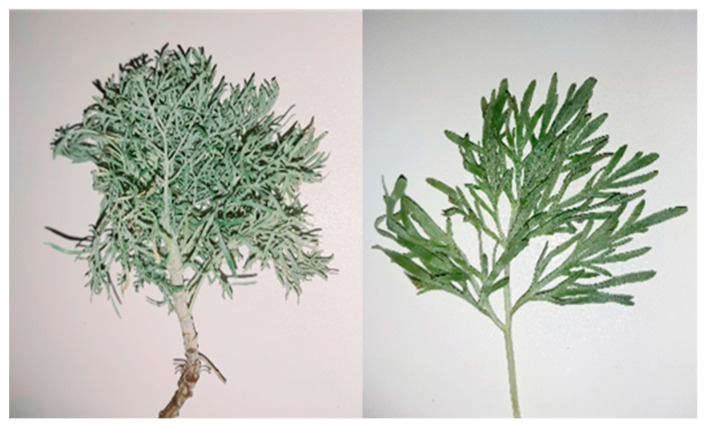
*A. absinthium* herb.

*Artemisia absinthium* L. (Figure 1) was collected in December 2020 in Zkara, a rural Moroccan commune of Mestferki, in the prefecture of Oujda Angad, in the eastern region of Morocco. It is located approximately 25 km from the city of Oujda. The leaves of *A. absinthium* were collected fresh and dried in a dry, airy place, away from light and sunlight. To obtain the ethyl acetate extract, the leaves (100 g) were extracted using a mixture of acetone/water (70/30) under reflux for 2 h, and the resulting solution was then filtered and concentrated by evaporation to 1/4 of the initial volume by using a rotary evaporator under reduced pressure at 40 °C. The extract obtained was fractionated with solvents of increasing polarity: hexane, diethyl ether, and finally, with an extraction of ethyl acetate to obtain the ethyl acetate extract (EAE). The aqueous extract was obtained by using five successive extractions of *A. absinthium* leaves with a Soxhlet extractor using five solvents (hexane, dichloromethane, ethyl acetate, acetone, ethanol); the sixth extraction is removed by distilled water to obtain the aqueous fraction (AQE); this technique was used to allow a minimum of molecules in this extract.

Extract yields (Y) were determined as the ratio between the mass of the extract obtained and the initial mass of the dried plant.

(1) $$Y\left(\%\right)=\frac{\mathrm{Mext}}{\mathrm{Mdp}}\times 100$$

where Mext and Mdp are extract mass and dry plant mass, respectively.

### 2.3. Content of Phenolic Compounds, Flavonoids, and Tannins

#### 2.3.1. The Total Polyphenol Quantification

The total polyphenol content in the *A. absinthium* extracts was determined according to the Folin–Ciocalteu method [35]. Indeed, 100 μL of extract solution with a concentration of 2 mg/mL was then mixed with 200 μL of Folin–Ciocalteu reagent in 2 mL of distilled water, and finally, 1 mL of sodium carbonate was added. (15%); the resulting mixture was incubated in the dark for 2 h at room temperature. Then the absorbance was measured at 765 nm using a spectrophotometer. The calibration curve is generated by using gallic acid according to a concentration range of (0–0.1 mg/mL). All experiments were performed in triplicate to take the mean of the experiments with the standard deviation. The amount of total phenolic compounds was expressed in mg gallic acid equivalents per gram of dry extract (mg GAE/g DE).

#### 2.3.2. The Flavonoids Quantification

The total flavonoid content of the *A. absinthium* extracts was obtained by using a colorimetric test using aluminum chloride (AlCl_3_), according to the method of Natalizia Miceli et al. [35]. Next, 500 μL of each extract (2 mg/mL) was blended with 1.5 mL of MeOH, and then 100 μL of AlCl_3_ (10%) was added, as well as 100 μL of potassium acetate (1 M) with 2.8 mL distilled water. The absorbance measurement was performed after 30 min of incubation at room temperature in the dark against a white solution at 415 nm. To determine the calibration curve, quercetin was used; a concentration range was established separately with quercetin (0–0.1 mg/mL). The results were expressed in mg quercetin equivalent per gram of dry extract (mg QE/g DE). All measurements were determined in three independent experiments to ensure the reproducibility of the results.

#### 2.3.3. The Condensed Tannin Quantification

The content of condensed tannin in the *A. absinthium* extracts was determined by the vanillin method according to the instructions of Mohti et al. [36]. First, 50 μL of the extract solution was mixed with 1.5 mL vanillin (MeOH, 4%) and 750 μL of concentrated acid (HCl) was added. The absorbance of the resulting mixture was measured at 500 nm after 20 min of incubation in the dark and at room temperature. Catechin was used to establish the calibration curve. The content of the condensed tannins was expressed in mg catechin equivalents per gram of dry extract (mg CE/g DE) and each extract was analyzed in triplicate.

### 2.4. High-Performance Liquid Chromatography Analysis

Our HPLC/DAD equipment comes from the Waters Corporation in the United States, the analysis of the extracts was performed using a liquid chromatography separation module (Waters e2695) coupled with a diode array detector (Waters 2998 PDA) and data were processed using Empower data processing software. The chromatogram was recorded at a wavelength of 254 nm to 300 nm. AC18 column (4.6 mm × 250 mm, 5 μm), a gradient mode of the mobile phase, was used. Solvent A is a mixture of ultrapure water/acetic acid (2% *v*/*v*) and solvent B is acetonitrile: 0 to 5 min: 95% A and 5% B, 25 to 30 min; 65% A and 35% B, 35 to 40 min; 30% A and 70% B, 40 to 45 min; 95% A and 5% B. The flow rate is 0.9 mL/min, and the injection volume is 20 μL. Peaks were identified by comparing their retention times and UV spectra with the standards used. The standard polyphenolic compounds used were caffeic acid, gallic acid, catechin, *p*-hydroxy benzoic acid, vanillin, naringenin, *p*-coumaric acid, ferulic acid, rosmarinic acid, rutin, vanillic acid, ascorbic acid, quercetin, trans-chalcone, malic acid, syringic acid, and kaempferol, which have all been previously found in *A. absinthium*.

### 2.5. Antioxidant Activity

#### 2.5.1. Scavenging 2, 2-Diphenyl-1-picrylhydrazyl Radical Test

The antioxidant activity of *A. absinthium* leaf extracts was determined by the method based on the decoloration of the DPPH (1,1-diphenyl-2-picrylhydrazyl) radical according to the protocol described by Miceli et al. [37]. A volume of 500 µL of each extract solution at different concentrations (from 0.0625 to 2 mg/mL) was added to 3 mL of the freshly prepared DPPH methanolic solution (0.1 mM). The negative control was made by mixing 500 µL of the solvent used for the solubilization of the extracts with a 3 mL solution of DPPH prepared in methanol. The absorbance value of the decolorization of the DPPH solution was read against a blank solution at a wavelength of 517 nm after an incubation time of 20 min at room temperature and in the dark using a UV/visible spectrophotometer. The positive control was represented by ascorbic acid, whose absorbance was determined under the same circumstances and with the same procedure as for the samples and for each concentration, and the assay was repeated three times. The scavenging activity was determined by the formula below:

(2) $$\mathrm{Radical}\;\mathrm{scavenging}\;\mathrm{activity}\;\left(\%\right)=\frac{\mathrm{Adpph}-\mathrm{As}}{\mathrm{As}}\times 100$$

where Adpph is the absorbance of the negative control, and As is the absorbance of the solution which contains the extract.

#### 2.5.2. The Ferric Reducing Power Assay (FRAP)

The reducing power of *A. absinthium* extracts was determined by the spectrophotometric method described by Miceli et al. [37], with modifications. Next, 0.5 mL of the different concentrations of the extracts solubilized in the suitable solvent (0.0625–2 mg/mL) were added to 1.25 mL of the phosphate buffer solution (0.2 M, pH 6.6) and to 1.25 mL of solution of potassium ferricyanide [K_3_Fe(CN)_6_] (1%). The mixtures were incubated at a temperature of 50 °C for 20 min. Afterward, 1.25 mL trichloroacetic acid solution (10%) was then added. The resulting mixture was then centrifuged at 3000 rpm for 10 min. In the end, 1.25 mL of the supernatant of each of the resulting solutions was added to the solution of 1.25 mL of distilled water and 0.25 mL of FeCl_3_ (0.1%). The absorbance was determined at a wavelength of 700 nm at room temperature after 10 min of incubation. The increase in the reaction medium absorbance indicated an increase in iron reduction. The positive control used was ascorbic acid. To ensure reproducibility, the results are the mean of three separate experiments.

#### 2.5.3. β-Carotene Bleaching Test

The antioxidant capacity of the plant extracts using the carotene bleaching test was measured using the following procedure from Kartal et al. [38]. The emulsion of β-carotene/linoleic acid was prepared by solubilizing 6 mg of β carotene in 1.5 mL of chloroform, 30 µL of linoleic acid, and 250 mg of Tween 80. The chloroform was evaporated using a rotary evaporator, and then 100 mL of distilled water saturated with oxygen H_2_O_2_ (30%) was added. The emulsion obtained was stirred to homogenize it. Next, 175 µL of extract solution or a reference antioxidant solution (BHA) at a concentration of 2mg/mL were added to 1.25 mL of the previous emulsion. The decoloration kinetics of the emulsion of the negative control and the extracts, or the BHA was followed at 490 nm for 120 min at regular time intervals. The relative antioxidant activity of the extracts (RAA) was determined according to the formula below:

(3) $$\mathrm{RAA}\left(\%\right)=\frac{A120-C120}{C0-C120}\times 100$$

where A120 is the absorbance of the solution which contains the extract after 120 min of incubation; C120 and C0 are the absorbances of the control after and before 120 min of incubation, respectively.

### 2.6. Inhibition of Carbohydrates Hydrolase Enzymes, In Vitro

#### 2.6.1. Pancreatic α-Amylase

The inhibition of the activity of the pancreatic α-amylase by the *A. absinthium* extracts was studied following the experimental protocol reported by Nour Elhouda Daoudi et al. [39]. The mixtures tested contained 200 mL of pancreatic α-amylase enzyme solution (13 IU), with 200 mL of the phosphate buffer (0.02 M; pH = 6.9), and an additional 200 mL of extracts of *A. absinthium* or acarbose for concentrations of 0.45 mg/mL and 0.9 mg/mL. The resulting mixtures were pre-incubated for 10 min at 37 °C. Then 200 mL of starch (1%) dissolved in the phosphate buffer was added and incubated at 37 °C for 20 min. To stop the enzymatic reaction, 600 mL of a colored DNSA reagent was added. Immediately after the addition of this reagent, the tubes were incubated a third time at 100 °C for 8 min and then placed in an ice-water bath for a few minutes. Finally, 1 mL of distilled water was added to dilute the resulting solution and the absorbance was measured at a wavelength of 540 nm. The percentage of pancreatic α-amylase enzyme inhibition was determined using the formula below:

(4) $$\mathrm{Inhibitory}\;\mathrm{activity}\;\mathrm{percentage}=\frac{\mathrm{Atest}\;540\;\mathrm{nm}-\mathrm{Acontrol}\;540}{\mathrm{Atest}\;540\mathrm{nm}}\times 100$$

where (Atest 540 nm) and (Acontrol 540 nm) were the absorbances of the solutions containing the extracts and without the extracts, respectively, at a wavelength of 540 nm.

#### 2.6.2. Intestinal α-Glucosidase

*A. absinthium* extracts have been tested for their ability to inhibit intestinal α-glucosidase activity according to the protocol described by Nour Elhouda Daoudi et al. [39], which consists of controlling the release of glucose obtained from sucrose degradation. The test solutions contained 100 mL of sucrose (50 mM) in addition to 1000 mL of the phosphate buffer (50 mM; pH = 7.5), and 100 mL of the intestinal α-glucosidase enzyme solution (10 IU). Next, 10 mL of the control (distilled water), positive control (acarbose), or solutions containing the *A. absinthium* extract at the concentrations of 165 and 328 mg/mL, respectively, were added to the previous mixture. Then the whole was incubated in a water bath at 37 °C for 25 min. The resulting solution was then heated for 5 min at 100 °C to stop the enzyme reaction. Finally, the glucose liberation was estimated using the glucose oxidase method with an automatically accessible kit (Glucose oxidase peroxidase). The absorbance of the final resulting solution was measured at 500 nm. The percentage of inhibition was determined by the formula given below:

(5) $$\mathrm{Inhibitory}\;\mathrm{activity}\;\mathrm{percentage}=\frac{\mathrm{Acontrol}\;500\;\mathrm{nm}-\mathrm{Atest}\;500}{\mathrm{Acontrol}\;540\;\mathrm{nm}}\times 100$$

where (Atest 500) and (Acontrol 500) were the absorbances of the solutions containing the extracts and without the extracts, respectively, at a wavelength of 500 nm.

### 2.7. Inhibition of Carbohydrates Hydrolase Enzymes, In Vivo

#### 2.7.1. Animals

The Wistar rats, weighing between 150 g and 250 g, used in this work were taken from the animal house of the Department of Biology, Faculty of Science, Mohammed First University, Oujda, Morocco. The rats were housed in animal cages, with access to water and food. They were kept in a well-ventilated room at a temperature of 24 ± 2 °C with a 12 h light/12 h dark cycle. The rats were treated and cared for according to the guide published by the United States National Institutes of Health (1985), which is internationally recognized for the proper treatment and care of laboratory animals.

#### 2.7.2. Induction of Diabetes

Diabetes was provoked in the rats by following the procedure described by [39]. The animals fasted for 16 h with ad libitum accessibility to water. They were then given an intraperitoneal injection of a unique dose of alloxan (140 mg/Kg) dissolved in a sodium-citrate buffer (pH = 3), cold and freshly prepared. One week later, rats with blood glucose levels above 1.5 g/L were chosen for use in this study.

#### 2.7.3. Pancreatic α-Amylase

Healthy and diabetic Wistar rats that had fasted for 16 h were used in this study. The animals tested were separated into four groups (*n* = 6; male/female = 1). The rats in the control group were treated with distilled water only (10 mL/Kg; po). The positive group or the rats were received the Acarbose (10 mg/Kg; po). Two treated groups received EAE or AQE for a dose of (200 mg/K; po). Then, 30 min of the administration of the solution, the rats of the different groups were orally loaded with starch (2 g/Kg). Afterward, by employing the glucose-peroxidase method, the blood glucose levels of the rats were estimated at 0, 30, 60 and 120 min.

#### 2.7.4. Intestinal α-Glucosidase

Healthy and diabetic Wistar rats used in this study were treated in the same way [40]. After fasting for 16 h, they were divided into four groups (*n* = 6; male/female = 1). The rats in the control group were treated with distilled water only (10 mL/Kg; po). The positive group received acarbose (10 mg/Kg; po). Group 1 received EAE (250 mg/Kg; po) and group 2 received AQE (250 mg/Kg; po). Then, 30 min of oral administration of the test substances, the rats of the different groups were loaded orally with sucrose (2 g/Kg). Blood samples were taken from the rats’ tails at different times under light anesthesia. Finally, the blood glucose levels of the rats were estimated by employing the glucose-peroxidase method for t = 0, 30, 60, and 120 min.

### 2.8. Statistical Analysis of Results

The results carried out are presented in the form of means ± standard errors, then they were statistically analyzed using computer software (Graph Pad Prism 5.04-San Diego, CA, USA). Comparative analysis between multiple groups was done by one-way variance analysis (ANOVA) and the statistical significance was accepted as *p* ≤ 0.05.

## 3. Results and Discussion

### 3.1. Yields, Phenols, Flavonoids, and Tannins Contents

Polyphenols are widely present in almost all medicinal and aromatic plant species; they are therefore an indispensable part of the human diet because of their health-promoting and antioxidant properties [41]. The total phenolic, flavonoid, and condensed tannin content, along with the extraction yields of EAE and AQE of *A. absinthium,* were examined and the results are presented in Table 1. The polyphenol content was calculated from the calibration curve of gallic acid (R = 0.998). EAE had the highest concentration of phenolic compounds (69 mg AGE/g DE), compared to AQE (31.534 ± 0.408 mg AGE/g DE). We obtained extraction yields of 15.95% for EAE and 0.672% for AQE. The flavonoid content of the *A. absinthium* extracts calculated from the quercetin calibration curve (R = 0.998), gives the highest content observed for EAE, with a value of 69.02 ± 0.33 mg QE/g DE vs. 31.534 ± 0.408 mg QE/g DE for AQE. For the condensed tannin (another class of bioactive compounds) [42], the results were calculated from the catechin calibration curve (R = 0.995) and contrarily to the previous results, AQE recorded the highest tannin content (3.070 ± 0.022) compared to EAE (0.987 ± 0.078). EAE showed a high content of polyphenols and flavonoids, while the AQE had a higher content of condensed tannin.

The values of the phenolic compound contents found in our extracts are lower than those obtained by Boudjelal et al. [43] (180.33 ± 16.25 mg GAE/g DE). On the other hand, the values obtained from the present study are higher than those found by Kružinauskaitė et al. [44], whose phenolic compound content of *A. absinthium* extracts varies from 21.19–24.74 mg GAE/g ED.

Polyphenols are one of the most common classes of secondary metabolites in flowering plants, as well as in fruits and flowers. These compounds are considered essential in the defense of plants against interferences and predators [45]. They are composed of one or more aromatic rings with one or more hydroxyl groups, along with other substituents. The presence of hydroxyl groups makes polyphenols very reactive in the neutralization of free radicals by giving a hydrogen atom or an electron, as well as chelate metal ions, thus reducing their pro-oxidant activity [46,47]. They have received the greatest attention for their effects in the treatment and prevention of several diseases. It has been proved that this type of molecule has numerous cardio-protective functions [48]; they can also prevent or delay depression, anxiety, and other diseases related to oxidative stress [49]. Several studies have indicated that polyphenols and their derivatives have shown anticancer capacities and an antioxidant potential for animal and human cervical cancer cells [50]. These compounds also can react with free radicals by stopping their activity, modulating the expression of genes involved in metabolism, thus repairing and protecting DNA damage, in addition to their ability to act as signaling molecules enhancing antioxidant defense [51].

Flavonoids are a class of phenolic compounds that are widespread in the plant kingdom [45]; many flavonoids are responsible for the attractive colors of leaves, fruits, and flowers [52]. Flavonoids are more appropriately called “nutraceuticals” because of the diversity of their pharmacological activities in the body. These elements have a powerful free radical scavenging capacity, as well as the advantage of being easily absorbed in the intestine after ingestion with minimal side effects and low toxicity in animals [53]. Flavonoids have many health benefits, thanks to the strength of their in vivo and in vitro antioxidant capacity [54,55]. Tannins are high molecular weight polyphenols that react with proteins when not oxidized [56]. These compounds are, in many cases, bioactive in plants [57]. They exist mainly in two forms: hydrolyzable or condensed [56]. Tannins, in turn, are endowed with antioxidant power. Thus, they inhibit superoxide formation, and hydrolyzable tannins inhibit lipid peroxidation [58]. Proanthocyanidins, or condensed tannins, are polymeric and oligomeric derivatives of the flavonoid biosynthetic process; they are increasingly recognized for their beneficial effects on health [59]. The quantity of tannin contained in the extracts is lower than that of the other bioactive components; however, their presence in the extracts cannot be ignored [60]. The degradation products of condensed tannins are potentially toxic to ruminants [61] and are absorbed by the small intestine of animals [62].

Based on our study outcome, it has been shown that polyphenols and flavonoids are most likely to be extracted using a solvent of average polarities, such as ethyl acetate, and that tannins can be obtained using a solvent with high polarity, such as water.

### 3.2. High-Performance Liquid Chromatography HPLC

**Figure 2:**
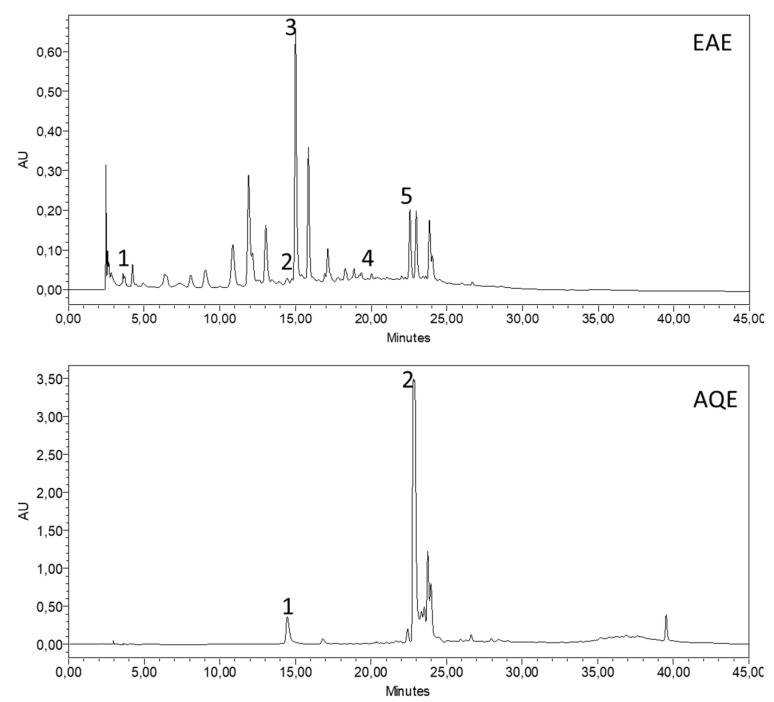
The HPLC chromatogram patterns of extracts from of *A. absinthium*: EAE (1) quercetin (1.29%), (2) *p*-hydroxybenzoic acid (0.21%), (3) caffeic acid (21.49%), (4) *p*-coumaric acid (0.77%), and (5) naringenin (5.85%); AQE (1) *p*-hydroxybenzoic acid (5.44%) and (2) naringenin (58.76%). EAE— ethyl acetate extract; AQE—aqueous extract.

**Figure 3:**
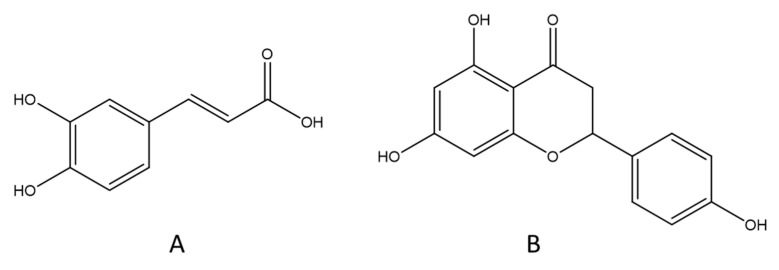
The structures of caffeic acid (**A**) and naringenin (**B**).

The chemical components contained in the *A. absinthium* extracts were analyzed using high-performance liquid chromatography coupled with a diode array detector (HPLC-DAD) by comparing their retention times and UV spectra to those of the standards. The HPLC chromatograms for the identified polyphenolics are presented in Figure 2. Figure 3 shows the chemical structure of the majority compounds found in AQE and EAE. The most abundant phenolic compounds were caffeic acid (21.49%) in EAE and naringenin (58.76%) in AQE. Other compounds were identified in these two extracts of *A. absinthium*, but at low percentages, namely *p*-hydroxybenzoic acid (5.44%) in AQE and quercetin (1.29%), *p*-hydroxybenzoic acid (0.21%), *p*-coumaric acid (0.77%) and naringenin (5.85%) in EAE. Similar findings were reported in the study by Lee et al. [63], with a total of 20 polyphenolic compounds in *A. absinthium* leaves, including hydroxybenzoic acids, hydroxycinnamic acids, flavanols, and caffeic acid.

The use of multiple successive extractions causes the AQE to be of low chemical composition, which is justified by a limited number of peaks; the solvents used successively in the extractions are of different polarity, and each solvent can selectively extract certain compounds depending on specific parameters, which therefore leads to the weakening of the ability of water, which is the sixth solvent used, to extract the previously extracted compounds; for EQA, the use of three liquid-liquid extractions decreases the extraction selectivity of the solvent ethyl acetate. These methods, therefore, consist of using extracts or fractions with a limited number of chemical compounds, making it possible to identify the element that is likely responsible for the observed activity.

Moacă et al. [64] indicated the presence of chlorogenic acid as the major product; quercitrin, rutin and isoquercitrin were also detected in lower concentrations, while *p*-coumaric acid, luteolin, gentisic acid, caffeic acid, and apigenin were also detected in trace amounts. Ivanescu et al. [65] detected the presence of ferulic acid and kaempferol in the unhydrolyzed extract, while the presence of *p*-coumaric acid, ferulic acid, fisetin, and patuletin was found in the extract of *A. Absinthium* after acid hydrolysis. Lee et al. [63] revealed the predominance of salicylic acid in the leaf extract, along with myricetin, caffeic acid, gallic acid and ferulic acid. The methanolic extract revealed the presence of chlorogenic acid as the main product [66]. Some works have reported the presence of gallic acid, caffeic acid [67], vanillic acid [68], vanillin, and naringenin [63] in *A. absinthium*. The studies of the chemical composition of *A. absinthium* yield different results, as the composition varies both qualitatively and quantitatively according to intrinsic factors related to the plant, the type of soil and climate, the periods of harvest, and the maturity of the plant, along with extrinsic factors related to the technique, duration, and temperature of the extraction, as well as the environment. 

Caffeic acid is a hydroxycinnamic acid derived from plants, and it is produced by various plant species as a secondary metabolite [69]. It is well known for its antioxidant activity; it has a direct primary function of scavenging harmful reactive species by electron transfer or hydrogen donation [70,71], a second function of chelating transition metals, especially iron, and copper [72] and an additional action of activating the redox-sensitive enzymes, improving the cytoprotective response [73]. This molecule has anti-diabetic properties [74].

Naringenin (flavonoid), a molecule that is considered biologically active, has positive bioactivities in diabetes, including hypoglycemia and antioxidant properties [75,76]. Indeed, naringenin may reduce renal glucose reabsorption and glucose adsorption by the brush border of the intestine, as well as increase glucose absorption and utilization by muscle and fat tissues [77], making it an excellent candidate for utilization in medicine as a treatment for type 2 diabetes in the fight against of insulin resistance. This molecule also has the advantage of low toxicity [77]. In addition, it is known for its antioxidant activity [78]. Naringenin reduces blood glucose and restores body weight, while normalizing serum lipid concentrations and the biomarkers of oxidative stress in the pancreas and liver, thus granting it potential as an anti-diabetic compound for future drug production [79].

### 3.3. Antioxidant Activity of A. absinthium Extracts

Phenolic compounds work as antioxidants; they have a strong ability to trap free radicals through their hydroxyl groups, which could serve as a basis for screening for antioxidant activity [80]. Flavonoids and condensed tannins also act as antioxidants as a result of their free hydroxyl groups.

In this study, we tested the DPPH free radical scavenging activities of EAE and AQE of *A. absinthium*. The free radical scavenging activity of the tested extracts is expressed as IC_50_ value (mg/mL) and the results are given in Table 2. The results indicate that EAE illustrates higher antioxidant activity (IC_50_ = 0.167 ± 0.004 mg/mL) than AQE (IC_50_ = 0.352 ± 0.019 mg/mL). The IC_50_ value of EAE is very close to that of ascorbic acid (IC_50_ = 0.158 ± 0.003 mg/mL).

**Figure 4:**
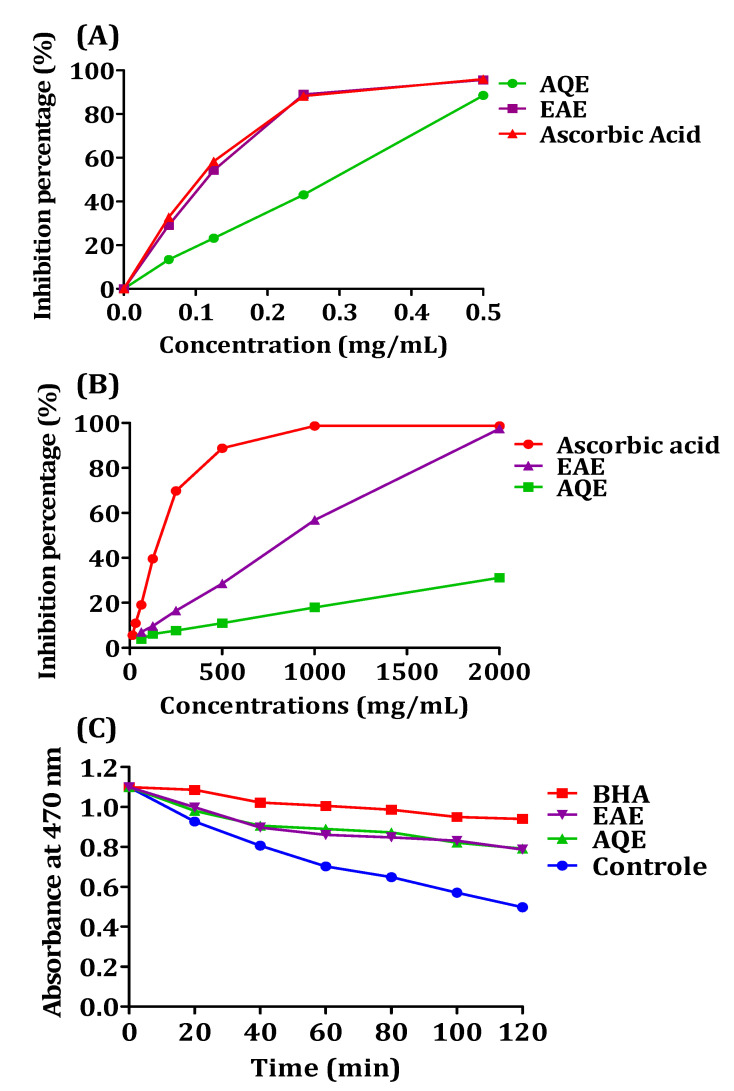
The activity of the scavenging DPPH radical, (**A**) iron reduction, (**B**) and bleaching kinetics of β-carotene, (**C**) in the presence and absence of EAE, AQE, and reference. EAQ—ethyl acetate extract; AQE—aqueous extract.

In the iron-reducing power test, the plant extracts induced a reduction of Fe^3+^ to Fe^2+^, which can be observed by appearance, as well as by measuring the absorbance of the blue coloration formed at 700 nm. Figure 4A shows the curves presenting the reducing powers of the *A. absinthium* extracts, and IC_50_ values are presented in Table 3. The reducing power sequence occurs in the following order: ascorbic acid > EAE > AQE. The reducing power of EAE and AQE increased from 0.249 ± 0.005 and 0.137 ± 0.007, respectively, at 0.062 mg/mL to 3.461 ± 0.041 and 1.107 ± 0.05 at 2 mg/mL, respectively.

The capacity of *A. absinthium* extracts to stop or retard lipid peroxidation was evaluated using the bleaching method of the β-carotene molecule, and the results are shown in Figure 4B. The results of relative antioxidant activity are shown in Table 3, and these results indicate that AQE and EAE were able to delay the decoloration of beta carotene by a similar inhibition of 48.7% and 48.3%, respectively.

The antioxidant capacity of *A. absinthium* extracts using the DPPH test is higher than that obtained by Moacă et al. [64] (IC_50_ = 0.4993 ± 0.0201 mg/mL), and by Craciunescu et al. [67] (IC_50_ = 0.57 ± 0.05 mg/mL), and lower than the results given by Msaada et al. [81], with IC_50_ values ranging from 9.38 ± 0.82 to 0.044 ± 1.92 mg/mL, contrastingly. The results for EAE are in accordance with those of Sidaoui et al. [82], who demonstrated that methanol–water extracts have powerful antioxidant capacities (IC_50_ = 0.118 mg/mL)**.** It should be noted that the chemical characteristics of the solvents used during extraction have a remarkable effect on the organic type of the agent’s antioxidants, resulting from the extraction process [83]. This difference in antioxidant activity may also be related to the differences in the content of the phenolic compounds, which differ according to several parameters.

From our results, we found a considerable correlation between phenolic compounds in both the DPPH and FRAP assays; these two techniques allow us to conclude that EAE presents a higher antioxidant activity than AQE. The antioxidant activity of EAE can be justified by the existence of polyphenolic compounds, as reflected by the high phenolic content. It is known that polyphenols exhibit considerable antioxidant activity. Nabavi et al. have found that increasing flavonoid content in the diet may reduce some human diseases [84].

The EAE and AQE of *A. absinthium* leaves showed similar and promising results in the β-carotene test, which was related to the polyphenols and tannins content in the two extracts. The reducing capacity of the plant’s extracts may indicate their potentially significant antioxidant power, which is usually related to the presence of reductones [85]. The antioxidant function prevents the formation of peroxide due to the presence of reductones that have the power to break the chain of free radicals by donating a hydrogen atom, or by reacting with certain peroxide precursors [86,87]. The free radical of linoleic acid is attacked by β-carotene so that it undergoes rapid bleaching as it loses the double bonds, and therefore, its orange color [88]; the existence of a molecule that plays the role of an antioxidant can inhibit or delay the destruction of the β-carotene molecule by neutralizing the radicals of the linoleic acid formed.

The presence of caffeic acid and naringenin as the major products in EAE and AQE, respectively, may be responsible for the observed antioxidant activities. Caffeic acid was found to have excellent antioxidant activity [89]; this activity was also shown to be stronger than that of Trolox and ascorbic acid [90], in a dose-dependent manner with an IC_50_ of 0.038 mg/mL [91]. Naringenin is a flavanone of natural origin, known for its effect on health, thanks to its antioxidant activity and its free radical scavenging capacity [92]. These two molecules may be the source of the antioxidant activity observed in EAE and AQE.

An excess of oxidants in the body can induce a phenomenon called oxidative stress, which is linked to the pathogenesis of several chronic diseases that can cause oxidative harm to proteins, DNA, and lipids. This process plays a key role in the development of several diseases that are the cause of most deaths today, including diabetes, cancer, atherosclerosis, eye disease, Alzheimer’s and Parkinson’s diseases [93], as well as other chronic diseases [93,94]. The intervention of antioxidants consists of the neutralization of the excess free radicals found in the organism, and therefore, an absence of oxidative stress responsible for the diseases. In fact, antioxidant substances have a strong capacity to trap free radicals. Antioxidants can be used in the cosmetic industry due to their ability to reduce oxidative damage, making them a good therapeutic alternative to prevent the premature onset of diseases [95]. The protective role of phytochemicals may be related to their antioxidant activity. The use of plant extracts rich in phenolic compounds appears to be a sustainable choice for cosmetic applications, ensuring a commitment to durability. Antioxidant compounds are used to inhibit the oxidation of the oil portion of cosmetic preparations and are also employed to reduce or prevent the oxidative deterioration of the active components of the product [96]. These compounds play a photoprotective role that helps in the treatment of sun-stressed or sensitive skin [97]. *A. Absinthium* has exhibited cytotoxic action on human colon and endometrial cancer cells, which may be due to its antioxidant power [66]. The methanolic extract of *A. absinthium* leaves was able to actively inhibit the proliferation of breast cancer cells with an IC_50_ value of 80.96 ± 3.94 μg/mL [98]. These studies suggest the use of this plant to develop new agents that can be used industrially or pharmaceutically, especially as anti-cancer agents.

### 3.4. Inhibitory Effect of Pancreatic α-Amylase Enzyme and Intestinal α-Glucosidase Enzyme, In Vitro

Due to the side effects and toxicity of the drugs currently used to control hyperglycemia, research has been directed at discovering new pancreatic α-amylase and intestinal α-glucosidase inhibitors from natural sources, especially plants that show a hypoglycemic effect with no or fewer side effects. Various plants that act as enzyme inhibitors have been tested for the management of diabetes. We evaluated the pancreatic α-amylase and intestinal α-glucosidase inhibitory activities of *A. absinthium* extracts. Acarbose, an oral hypoglycemic drug employed for treating diabetes mellitus, was used as a positive control [99].

**Figure 5:**
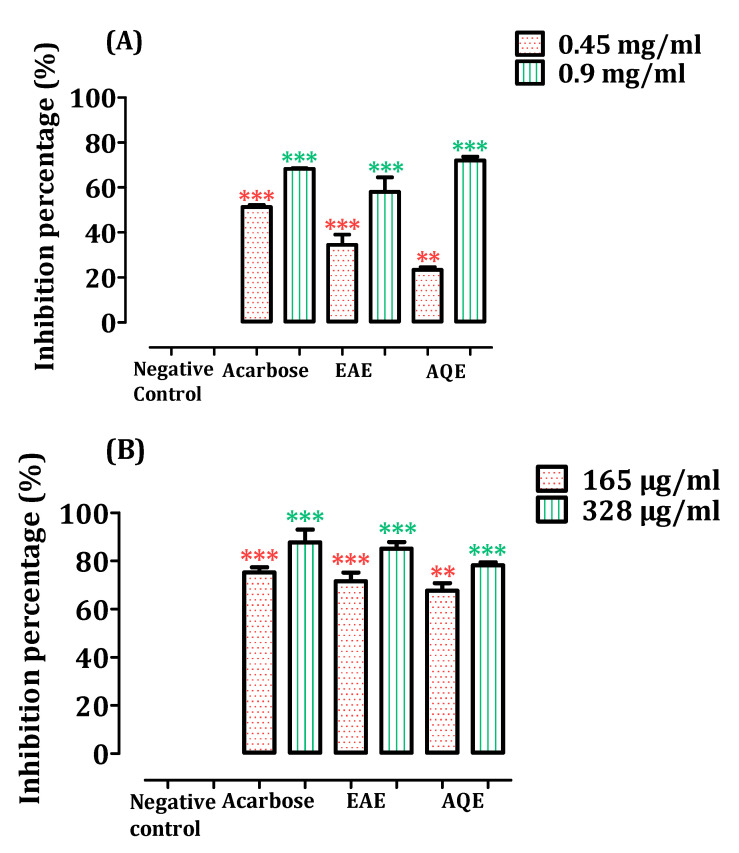
The effect of AQE and EAE of *A. absinthium* on pancreatic α-amylase enzyme (**A**), and intestinal α-glucosidase enzyme (**B**), inhibition in vitro (*n* = 3). ** *p* < 0.01; *** *p* < 0.001 as compared to the control. EAE—ethyl acetate extract; AQE—aqueous extract.

Figure 5A showed that both extracts of *A. absinthium* significantly inhibited the activity of pancreatic α-amylase enzyme, in vitro, in a nearly similar manner to that of the control. The concentration of 0.9 mg/mL showed a more potent inhibitory effect than 0.45 mg/mL concentration, with an inhibitory percentage of 58.14 ± 4.57% for EAE and 72.06 ± 1.17% for AQE. AQE and EAE inhibit the enzyme, as shown by the IC_50_ values of 0.68 ± 0.01 mg/mL and 0.76 ± 0.064 mg/mL, respectively (Table 3). Moreover, the statistical analyses showed that AQE possesses the same effect as EAE (*p* > 0.05).

The results of the inhibitory activity of the intestinal α-glucosidase enzyme in the extracts of *A. absinthium* are shown in Figure 5, revealing a considerable inhibition against this enzyme compared to the positive control. The results indicated that both EAE and AQE significantly inhibited the intestinal α-glucosidase enzyme activity (*p* < 0.001), in vitro, in comparison with the control, which shows a value of 0.155 ± 0.0009 mg/mL for EAE and 0.170 ± 0.002 mg/mL for AQE (Table 3). As compared with the positive control (acarbose), *A. absinthium* revealed statistically the same activity as the positive control at the concentrations of 0.165 mg/mL and 0.328 mg/mL. The ethyl acetate extract EAE appeared to be the most active extract, with an inhibition percentage of 85.1%, close to that of the positive control (acarbose), which has an inhibitory activity of 87.7 for a concentration of 0.328 mg/mL.

Oligosaccharides and disaccharides resulting after the hydrolysis of polysaccharides by pancreatic α-amylase are transformed into monosaccharides in the presence of the enzyme α-glycosidase, which are absorbed in the hepatic portal vein by the small intestine and subsequently, increase postprandial glycemia [22]. Therefore, the use of these extracts inhibits the formation of monosaccharides; consequently, there will not be an increase in blood glucose levels, indicating a hypoglycemic effect.

The activity of AQE shown for the inhibition of these two enzymes may be related to the presence of caffeic acid, which presents as the majority product after extraction. Indeed, Ganiyu Oboh et al. showed that caffeic acid displays an inhibitory effect on pancreatic α-amylase (IC_50_ = 0.003 mg/mL) and intestinal α-glucosidase (IC_50_ = 0.004 mg/mL) [91]. These IC_50_ values remain smaller than those of our extract, and we can attribute this result to the antagonism effect of caffeic acid in the presence of other molecules.

For many years, plants were considered as the main source of drugs and oral hypoglycemic agents, so research has been directed towards the discovery of those plants possessing pancreatic α-amylase and intestinal α-glucosidase inhibitory power, and thus with potential for use as hypoglycemic agents. Among the plants used as inhibitors of pancreatic α-amylase and intestinal α-glucosidase are *Andrographis paniculata* [100], *Hibiscus sabdariffa* Linn. [101], *Morinda lucida* Benth [102], *Telfairia occidentalis* [103], *Phaseolus vulgaris* L. [104] and *Andromachia igniaria* [105].

The *Asteraceae* family is considered one of the most common anti-diabetic plant sources in traditional Moroccan medicine [106], and artemisia is a large and diverse genus of plants in the *Asteraceae* family. In the last few years, there has been a growing focus on the research of bioactive components of artemisia. Some plants belonging to this genus have shown a capacity for the inhibition of the pancreatic α-amylase enzyme and the intestinal α-glucosidase enzyme, among these plants we find Artemisia anethifolia, Artemisia desertorum, Artemisia latifolia, Artemisia umbrosa, Artemisia tanacetifolia, Artemisia palustris, Artemisia leucophylla and Artemisia commutata [107].

### 3.5. Inhibitory Effect of Pancreatic α-Amylase Enzyme and Intestinal α-Glucosidase Enzyme, In Vivo

**Figure 6:**
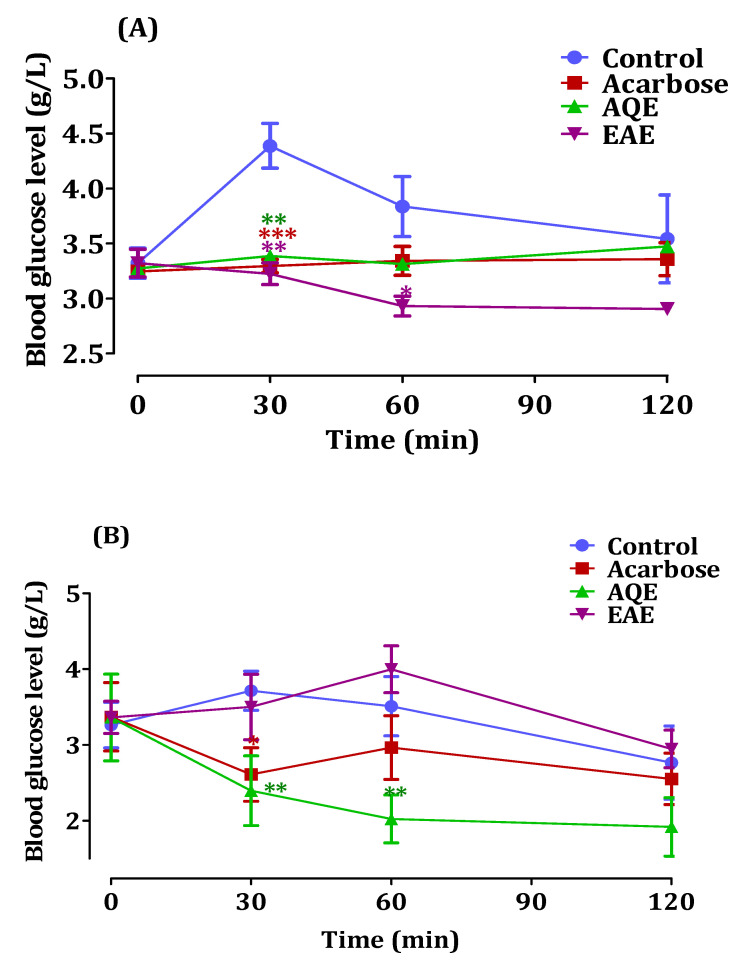
The effects of *A. absinthium* extracts and acarbose on glycemia in normal rats after starch overload (**A**), and after sucrose overload (**B**) (*n* = 5) ((* *p* < 0.05), (** *p* < 0.01) and (*** *p* < 0.001)).

To confirm the inhibitory activity of AQE and EAE extracts of *A. absinthium* on pancreatic the α-amylase enzyme and intestinal α-glucosidase enzyme, in vivo, the test was performed and the results are presented in Figure 6. Figure 6A represents the effect of *A. absinthium* extracts on blood. After oral starch overload in normal rats, the results indicated that the blood glucose level in the control group increased from 0.81 g/L to 1.32 g/L after 30 and 120 min of starch administration, respectively. While in the presence of AQE and EAE, the postprandial blood glucose decreased compared to the control from 1.84 g/L to 0.60 g/L and from 0.82 g/L to 0.62 g/L, respectively, after 120 min. However, EAE and AQE showed significantly higher activity than acarbose at a dose of 10 mg/Kg.

Figure 6B demonstrates the results of glucose level monitoring in normal rats. The blood glucose level increased to 1.65 g/L and 1.22 g/L during 120 min in the control group, whereas in the presence of the aqueous extract, blood glucose levels decreased from 1.26 g/L to 0.99 g/L 120 min after sucrose administration, respectively. EAE showed no significant anti-hyperglycemic effect of this enzyme in the normal Wistar rats; in fact, it shows a similar effect to that of the control.

**Figure 7:**
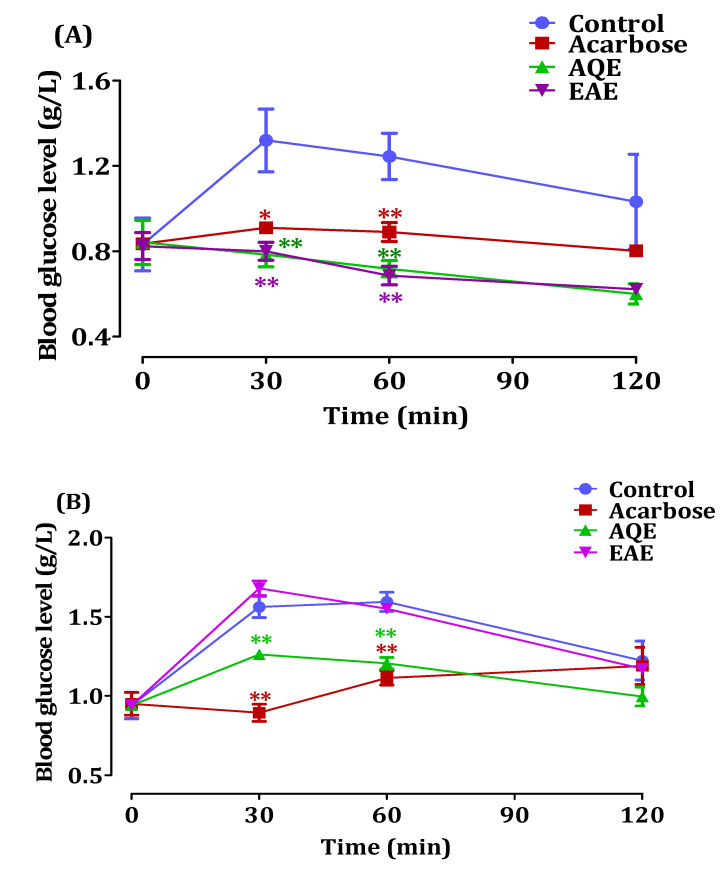
The effect *A. absinthium* extracts and acarbose on glycemia in diabetic rats after starch overload (**A**), and after sucrose overload (**B**) (*n* = 5) ((* *p* < 0.05) and (** *p* < 0.01)).

The results in Figure 7 correspond to the similar oral starch and sucrose tolerance test conducted previously, but this time on diabetic rats. The effect of *A. absinthium* extracts on blood glucose after oral starch overload is shown in Figure 7A. The results show that in the presence of EAE, postprandial blood glucose decreased remarkably 30 min after oral starch overload, decreasing to 3.22 and 2.9 g/L at 60 and 120 min, respectively. On the other hand, blood glucose levels increased by 3.67 g/L and 4.52 g/L in the control non-treated group. The group treated with AQE showed a decrease in blood glucose at both 30 and 120 min, with levels of 4.38 and 3.47 g/L, respectively. It can be seen that the blood glucose value in diabetic rats treated with AQE showed similar levels to those treated with acarbose after the oral starch overload; however, EAE showed a remarkably higher hypoglycemic effect than acarbose.

Figure 7B demonstrated the results of the oral sucrose tolerance test of *A. absinthium* extracts on streptozotocin-induced diabetic rats. In the control group, the level of glycemia increased from 3.26 to 3.71 g/l 30 min after sucrose overload, while, in presence of 250 mg/Kg of EAE, the glycemia level decreased (*p* < 0.01) from 3.36 to 2.39 g/L and from 3.36 to 2.024 g/L 30 and 60 min after sucrose overload, respectively. After 120 min, we found that the blood glucose level changed and became equal to the control values for all the tested groups. In the presence of EAE, the blood glucose levels revealed no significant variation from the control group (*p* > 0.05). Indeed, the blood glucose level increased from 3.36 g/L to 3.5 g/L 30 min after sucrose overload and to 3.99 g/L 60 min after; the blood glucose level decreased to 2.94 after 120 min. Li et al. [30] showed that *A. absinthium* exhibits antidiabetic activity in diabetic humans, with no significant effect on lipid profiles, while, Daradka et al. [31] showed that the ethanolic extract of *A. absinthium* has a hypoglycemic activity in rats with alloxan-induced diabetes. These results are in agreement with our work, which has confirmed the antidiabetic power of this plant.

α-amylase is one of the main enzymes in the human body, identified as either salivary amylase or pancreatic amylase, responsible for the degradation of starch into simple sugars. In general, this enzyme hydrolyzes polysaccharides to produce disaccharides and oligosaccharides. The inhibition of this enzyme by our extracts resulted in a decrease in the formation of oligosaccharides and disaccharides, causing a decrease in the absorption of glucose into the blood. The inhibition of pancreatic α-amylase and intestinal α-glucosidase enzymes has the potential to significantly decrease the postprandial rise in blood glucose; therefore, it may be an important management strategy for type 2 diabetic and borderline diabetic patients [108,109].

The in vivo antidiabetic activity found for AQE may be due to the main by-product after extraction, which is caffeic acid. Feng-Lin Hsu et al. demonstrated that caffeic acid reduced the elevation of plasma glucose levels in insulin-resistant rats subjected to a glucose challenge test. In addition, this molecule increased the glucose uptake in isolated adipocytes [74]. The antidiabetic activity found in AQE may be due to the presence of a high tannin content; indeed, tannins have shown positive effects in the treatment of diabetes mellitus type 2, and this effect is probably due to the inhibition of alpha-amylase thanks to its ability to bind carbohydrates and proteins and possibly also due to the inhibition of alpha-glucosidase [110,111].

The activity found in EAE of pancreatic α-amylase can be linked to the presence of naringenin, with a percentage of 58% in this extract. In fact, Osama M. Ahmed et al. have shown that naringenin has a potent antidiabetic effect in NA/STZ-induced type 2 diabetic rats, thanks to its insulin-enhancing property and its insulinotropic effect, which in turn may be facilitated by adiponectin expression in adipose tissue and the enhancement of the insulin receptor GLUT4 [112]. The activity of this extract in vivo on intestinal α-glucosidase did not show any hypoglycemic effect on rats; this result confirms that the in vitro inhibitory effect is not necessarily the same as the in vivo effect.

## 4. Conclusions

The abundance of *A. absinthium* in Morocco has led us to search for ways to utilize it. Our work has shown that the ethyl acetate extract and the aqueous form of *A. absinthium* leaves have antioxidant properties, in addition to being able to inhibit the pancreatic α-amylase enzyme and the intestinal α-glucosidase enzyme involved in the degradation of sugars, thus causing a hypoglycemic effect. This result is justified by the high content of polyphenols and by the high antioxidant activity found in the extracts. Ethyl acetate extract does not show any inhibitory activity on intestinal α-glucosidase in vivo, but it exhibits remarkable activity in vitro; hence, the importance of in vivo studies. This result is not justified, but it may be linked to the presence of one or more molecules which do not react with the animal’s enzymes. The presence of naringenin in the acetate extract and of caffeic acid in the aqueous fraction of *A. absinthium* could explain the inhibition activity of α-amylase pancreatic and the antioxidant properties found.

## Figures and Tables

**Table 1:** The yield of extraction and the contents of the total phenolic compounds, flavonoids, and condensed tannins in *A. absinthium* extracts.

	Yield (%)	Total Phenolic (mg GAE/g DE)	Flavonoid (mg QE/g DE)	Condensed Tannins (mg CE/g DE)
AQE	0.672 ± 0.088	31.534 ± 0.408	11.246 ± 0.184	3.070 ± 0.022
EAE	15.95 ± 0.252	69.013 ± 0.249	25.842 ± 0.241	0.987 ± 0.078

EAE—ethyl acetate extract; AQE—aqueous extract.

**Table 2:** The IC_50_ values were obtained for the DPPH and FRAP tests, and the RAA% for the β-carotene bleaching test.

	IC_50_ (mg/mL)		RAA %
	DPPH	FRAP	β-Carotene
BHA	-	-	73.4
Ascorbic acid	0.158 ± 0.003	0.137 ± 0.077	-
AQE	0.352 ± 0.019	3.361 ± 0.043	48.7
EAE	0.167 ± 0.004	0.923 ± 0.028	48.3

RAA—relative antioxidant activity; BHA—butylated hydroxyanisole; EAE—ethyl acetate extract; AQE—aqueous extract.

**Table 3:** The IC_50_ values of *A. absinthium* extracts and acarbose in pancreatic α-amylase and intestinal α-glucosidase inhibition.

	IC_50_ (mg/mL)	
	Pancreatic α-Amylase	Intestinal α-Glucosidase
Acarbose	0.58 ± 0.003	0.148 ± 0.002
EAE	0.68 ± 0.010	0.155 ± 0.0009
AQE	0.76 ± 0.064	0.170 ± 0.002

EAE—ethyl acetate extract; AQE—aqueous extract.

## Data Availability

Data are available upon request.
